# Contextual factors influencing physicians’ perception of antibiotic prescribing in primary care in Germany — a prospective observational study

**DOI:** 10.1186/s12913-022-07701-3

**Published:** 2022-03-12

**Authors:** Annika Queder, Christine Arnold, Michel Wensing, Regina Poß-Doering

**Affiliations:** Department of General Practice and Health Services Research, University Hospital Heidelberg, Heidelberg, Germany

**Keywords:** Rational antibiotic use, Primary care, Physicians’ decision-making, Context analysis

## Abstract

**Background:**

Antimicrobial resistance is a worldwide challenge for health services and systems alike. To reduce the overuse of antibiotics, multifaceted interventions are often used to achieve sustainable effects. It can be assumed that these effects are influenced by contextual factors. Embedded in the cluster randomized trial ARena (Sustainable reduction of antibiotic-induced antimicrobial resistance), the aim of this present study was to identify contextual factors associated with practitioners’ perceptions of antibiotic prescribing in German primary health care.

**Methods:**

In a prospective observational study, data were generated in a three-wave survey study between January 2018 and July 2019. Analysis was performed using logistic regression models. The outcome of interest was the physician perceived impact of participating in the ARena project on decision-making regarding antibiotic prescribing, the independent variables of interest included individual characteristics, intervention arm allocation, primary care network (PCN) environment and characteristics of the medical practice.

**Results:**

Forty Six point eight percent (*n* = 126) of participants indicated to have perceived an impact on their decision-making regarding antibiotic prescribing by participating in the ARena project. Bivariate logistic regression analyses indicated that work experience (odds ratio (OR) 1.05, 95% confidence interval (CI) 1.006–1.103), PCN environment (OR 2.06, 95% CI 1.256–3.363), structural conditions (OR 1.66, 95% CI 1.161–2.371), environment of existing processes (OR 1.46, 95% CI 1.011–2.094), and externally defined general conditions (OR 1.57, 95% CI 1.035–2.378) were associated with physicians’ perceived impact of participating in the ARena project on decision-making regarding antibiotic prescribing. In the multivariable logistic regression analysis, only work experience OR 1.05 (95% CI 1.001–1.104) continuously showed a significant influence.

**Conclusions:**

This study indicates that contextual factors at individual, practice, and system level influence physicians’ perceptions of antibiotic prescribing. Longer work experience appeared to be a significant influencing factor to be considered in antimicrobial stewardship programs.

**Trial registration:**

ISRCTN, ISRCTN58150046 (registered 13.09.2017).

**Supplementary Information:**

The online version contains supplementary material available at 10.1186/s12913-022-07701-3.

## Background

Antimicrobial resistance (AMR) is a worldwide challenge for health services and systems alike. The AMR rate is strongly correlated with the use of antibiotics in human medicine [1, 2]. A major proportion of antibiotic use is related to the primary healthcare sector, which emphasizes importance and need for research and action [2, 3].

In order to meet the challenge AMR poses for the general public and the healthcare system, the policy to curb antibiotic prescribing in human medicine has been strengthened in Germany by a national political agenda [1]. Similar to other countries, the primary care sector in Germany accounts for a large proportion of antibiotic consumption [1]. Although the use of antibiotics in Germany decreased from 13.4 defined daily dose (DDD) per 1,000 inhabitants per day in 2010 to 11.4 DDD per 1,000 inhabitants per day in 2019 [4] and antibiotic consumption is considered moderate compared to other European countries, there is still considerable potential for improvement [1, 5].

To meet the challenge, the ARena (Sustainable reduction of antibiotic-induced antimicrobial resistance) project was initiated to promote the rational and appropriate use of antibiotics for acute, non-complicated and self-limiting infections in primary care. Aiming to promote awareness and understanding of the growing challenges associated with AMR, ARena addressed knowledge and attitudes of primary care physicians, primary care teams as well as patients about the use of antibiotics, using a multifaceted strategy with multiple interacting intervention components [6]. Such complex multiple component interventions (CMCI) are increasingly used in health services research. Expected results include a fast progression of innovation and that a synergy of various effects could address the high complexity in healthcare [7, 8]. Particular interest is paid to intervention- and context-dependent factors that might cause changes [7].

Even though the concept of context is widely used in health services research, a consistent definition and application in practice still lacks [9]. In the present study, the term ‘context’ is understood as defined by Pfadenhauer et al. [10]: “reflects a set of characteristics and circumstances […] within which the implementation is embedded” and “interacts, influences, modifies and facilitates or constrains the intervention and its implementation” [10]. In this understanding, context is an overarching concept that encompasses physical location as well as relationships, roles and interactions at multiple levels [10].

The aim of this study was to identify contextual factors relevant to physicians’ decision-making in antibiotic prescribing in German primary healthcare. Data collected in a survey study conducted within the ARena process evaluation were used to explore whether primary care physicians perceived their participation in the ARena project to have an impact on their decision-making in antibiotic prescribing. Potential findings can inform design, tailoring and implementation of antimicrobial stewardship programs by identifying determinants and understanding mechanisms of action, thus achieving the uptake of research findings into routine healthcare, and improving healthcare services [7].

## Methods

To report this study, the “Strengthening the Reporting of Observational Studies in Epidemiology (STROBE-) Statement” checklist was used [11].

### Research design and conceptual framework

For this study, a prospective observational study design following an exploratory approach was used. ARena was a three-armed (non-blinded) cluster randomized trial conducted in 14 primary care networks (PCNs) in Bavaria and North-Rhine Westphalia with the aim of a sustainable reduction of antibiotic use in acute, non-complicated and self-limiting infections. Using a multifaceted strategy with multiple interacting intervention components, primary care physicians and teams in the three intervention arms (I, II, III) received a standard set of intervention components containing e-learning on communication, moderated quality circles including data-based feedback on antibiotic prescribing, performance-based additional reimbursement, public information campaigns, and culture-sensitive information material for patients in printed format. As add-on to this standard set, arm II received additional culture-sensitive information material for patients in digital format via tablet computers provided in the practices’ waiting areas. Intervention arm III received a computerized decision support system and additional interdisciplinary quality circles as add-on to the standard set. As participatory, problem-solving group approaches, all quality circles aimed to facilitate critical discussion of clinical practice and reflection of routines to promote care quality [12]. Randomization for the intervention study was done by statisticians at the Department of Medical Biometry, Institute of Medical Biometry, University Hospital Heidelberg. The evaluation research covers both an outcome and a process evaluation. Detailed descriptions of the project and the intervention components [6], the process evaluation, [13], and the outcome evaluation [14, 15] were published elsewhere. See Additional file 1 for description of the study design and the process evaluation design in ARena and a brief description of intervention components. For this study, data generated through the three-wave survey study within the ARena process evaluation were used. The study-specific survey questionnaires (T0, T1, T2) were developed based on the Theory of Planned Behavior (TPB) [16]. The TPB describes four central aspects to explain behavior: (1) attitude towards behavior, (2) subjective norms related to behavior, and (3) perceived behavioral control. These predict (4) behavioral intentions which in turn can lead to behavior [16]. The TPB as well as the Context and Implementation of Complex Interventions (CICI) framework, further scientific literature and specific factors derived from the project environment (such as membership in a PCN or the assignment to one of the intervention arms) were used to identify possible contextual factors underlying the analysis in this study. In the CICI framework, context comprises seven domains: (1) epidemiological, (2) geographical, (3) ethical, (4) socio-cultural, (5) socio-economic, (6) legal, and (7) political context [10]. Therefore, it was assumed that these contextual factors have a significant impact on a desired change in behavior. To understand which factors influence practitioners’ antibiotic prescribing patterns, it was important to understand how behavior occurs.

### Data collection

Data were collected by the study team at the Department of General Practice and Health Services Research at the University Hospital Heidelberg at three measurement points: T0 in January 2018 (3 months after start of intervention), T1 in October 2018, and in T2 July 2019 (directly after completion of intervention). The survey questionnaires related to participant engagement in the intervention and views on intervention components. In addition, T1 and T2 questionnaires also asked for intermediate and final participant assessment of the intervention components. All primary care physicians participating in the ARena project were invited to participate in the paper-based survey (T0 *n* = 303, T1 *n* = 312, T2 *n* = 292). To increase the response rates, E-mail reminders were sent out after four weeks each time.

Survey questionnaires comprised socio-demographic questions, items focused on implemented intervention components, and relevant contextual factors as well as prescribing decisions and general perceptions regarding antibiotics. An overview of all relevant questions for this analysis is provided in Additional file 2. For analysis, data from all completed questionnaires were digitalized and transferred into IBM SPSS Statistics 24. Findings are reported here with a focus on contextual factors of the ARena project and their impact on decision-making in antibiotic prescribing by primary care providers.

### Outcome

A 5-point Likert-Scale (completely disagree/disagree/neutral/agree/completely agree) was used to measure physicians’ perception of the impact participation in the ARena project had on decision-making regarding antibiotic prescribing in the response to the item “My decision-making to prescribe antibiotics is influenced by my participation in the ARena project” in T2. For bivariable and binary logistic regression analysis, the ordinal variable was modified to a dichotomy variable: ‘0’ = participation in the ARena project did not impact decision-making in antibiotic prescribing (completely disagree/disagree/neutral) and ‘1’ = participation in the ARena project did impact the decision-making in antibiotic prescribing (agree/ completely agree). The value ‘neutral’ indicated that participants were indecisive or could not attribute a clear impact of their study participation on their decision-making regarding antibiotics prescribing.

### Predictive factors

Possible influencing factors were examined based on the theoretical frameworks of the TPB and CICI, prior study findings and specific factors derived from the project environment. In this study, four domains of possible influencing factors were included: (I) individual characteristics, (II) intervention arm allocation, (III) PCN environment, and (IV) general characteristics of the medical practice.

(I): All variables of individual characteristics were measured at time of T0. Individual-level factors included in this study were sex (female or male), age and work experience (count variables), employment type (employed or independent), employment time (part time or full time), and medical specialty (free text response variable classified as dichotomous variable of ‘general practitioner or specialist’).

(II): To investigate the impact of the interventions on decision-making in antibiotic prescribing, the assignment to the intervention arm was tested and measured in a categorial variable with the values I, II and III.

(III): The context of PCN can be assigned to both, the socio-cultural and ethical context domain of the CICI and was measured with eleven 5-Point Likert-Scale items in T0 (completely disagree to completely agree). For this study, the respective mean value scores of the items were formed for all completely answered cases. The scale was validated with a Cronbach’s α coefficient of 0.898 (see Additional file 3). The mean value score was interpreted as a scale from 1 (not supportive) to 5 (very supportive), indicating how supportive participants consider their PCNs’ environment.

(IV): General conditions of medical practices were described by the number of patients per quarter of year classified as group 1 (< 500), group 2 (500–1,000) group 3 (1,001–1,500) and group 4 (> 1,500) and were re-coded in a dichotomous variable indicating practice size with group 1 (≤ 1,000) and group 2 (≥ 1,001) patients per quarter of year for this analysis. Practice catchment area was classified by five categories referring to population size (group 1 (< 5,000), group 2 (5,000 – < 20,000), group 3 (20,000 – < 100,000), group 4 (100,000 – < 500,000), group 5 (≥ 500,000)) and included in this analysis as dichotomous variable ‘population’ (< 100,000 and ≥ 100,000). These factors relate to the epidemiological and geographic context domains of the CICI. Further contextual factors such as “structural conditions” (practice personnel and rooms), “environment of existing processes in the practice” (routines of daily care) and “externally defined general conditions” (statutory care quality requirements or specifications for reimbursable services and remuneration by health insurers) were each polled with six 5-Point Likert-Scale items (completely disagree to completely agree). Mean value scores were calculated (scale was validated with a Cronbach’s α coefficient of 0.921/ 0.907/ 0.890) for the respective domains as a scale that indicates how supportive each context-domain is perceived by the participants (scale from 1 (not supportive) to 5 (very supportive)). For detailed survey items see Additional file 3. All items in this domain were measured in T0. In relation to the CICI, structural conditions are to be classified in the domains of the geographical and socio-economic context. The environment of existing processes in the medical practice is linked to the geographical and socio-cultural context domain, and externally defined general conditions are to be classified to the socio-economic, political and legal context domain of the CICI.

### Statistical analysis

IBM SPSS Statistics 24 (IBM, Armonk, NY, USA) was used for all analyses. Data visualization was performed using Microsoft Excel 2016 (Microsoft Corp., Redmond, WA, USA).

All variables were analyzed descriptively (absolute and relative frequencies for nominal scale levels and means with Standard Deviations (SDs) for continuous variables). Respondents’ baseline characteristics (T0) were summarized and a comparison between intervention arms was made using adequate tests depending on the type of variables (Phi/Cramer-V, Chi^2^, Eta coefficient, and Kruskal–Wallis (H-)Test for comparison of mean values).

Bivariate analyses were performed using cases answered at T0 and T2 between the dependent variable (measured in T2) and 10 predictive variables (measured in T0). Correlation tests (Cramer-V and Eta) and binary logistic regression models were performed to evaluate correlations between the predictive variables and the dependent variable. The Odds Ratio (OR) was calculated with a Confidence Interval (CI) of 95%.

Due to the small underlying number of cases (*n* = *152*), the inclusion of independent variables was limited [17]. Thus, to take the theoretical background of the CICI into account, four multivariable binary logistic regression models were performed separately to consider the influence of the predictive contextual factors in the four domains (1) individual characteristics, (2) intervention arm allocation, (3) PCN environment, and (4) general characteristics of the medical practice. Only statistically significant results (*p-value* ≤ *0.05*) were included in the further multivariable analyses.

A multivariable binary logistic regression model was employed to analyze the influence of the contextual factors on the perceived impact of participation in the ARena project on decision-making regarding antibiotic prescribing. The predictive factors were included stepwise to consider changes in coefficients.

The requirements for binary logistic regression were examined and multi-collinearity was tested.

The participants were nested in a total of 14 PCNs (2–27 per network). In order to take the clustered structure of the data into account, a Multilevel Analysis (MLA) was carried out for the PCNs. MLA is a method used in health services research to examine the associations between context in health care organizations and the objective [18, 19]. Therefore, the intra-cluster correlation coefficient (ICC) in multilevel logistic regression was calculated to examine the share of the between-cluster variance in the total variance [20]. Finally, a mixed logistic model with a random intercept defined by any given PCN was employed.

Missing values were analyzed descriptively and a Little’s Missing Completely at Random (MCAR)-Test was performed (*p* = *0.185*), showing that missing values were completely at random (see Additional file 4). The significance level for all analyses was set at 5%.

## Results

### Sample characteristics

Response rates were 75.5% (T0 *n* = 229), 64.0% (T1 *n* = 200) and 63.3% (T2 *n* = 184) [21]. At time of T0, 148 (55.0%) of all responders were male and the average age was 54.0 years (SD 7.6). 132 (49.1%) were general practitioners (GPs) and 95 (35.3%) were specialized physicians, including qualifications of internal medicine, otolaryngology, urology, paediatrics, pneumology and gynaecology. On average, participants had 25.5 years (SD 7.9) of work experience. 65 (24.2%) of them worked in a single practice, 154 (57.2%) in group practices, and 10 (3.7%) in shared rooms (separate financial entities and indemnity insurances, but sharing rooms, equipment and staff). 193 (71.7%) were self-employed. 90 (33.5%) of the participants worked full time. Table 1 shows the characteristics of the participating physicians at T0, differentiated according to the intervention arms. There were no statistically significant differences in relevant characteristics of participants between the intervention arms (see Additional file 5 Table 1).

**Table 1:** Characteristics of participants by intervention arm allocation (T0)

**Characteristics**	**Total** *n* = 229	**Intervention arm I** *n* = 76 (33.1%)	**Intervention arm II** *n* = 73 (32.0%)	**Intervention arm III** *n* = 80 (34.9%)
**Sex**	224 (100)	73 (100)	73 (100)	78 (100)
Female, n (%)	76 (33.9)	18 (24.7)	25 (34.2)	33 (42.3)
Male, n (%)	148 (66.1)	55 (75.3)	48 (65.8)	45 (57.7)
**Age**, n	221	72	73	76
mean (SD)	54.4 (7.6)	54.7 (7.5)	54.6 (8.3)	54.1 (7.1)
**Medical Speciality**	227 (100)	76 (100)	71 (100)	80 (100)
GP, n (%)	132 (58.1)	44 (57.9)	40 (56.3)	48 (60.0)
Specialists, n (%)	95 (41.9)	32 (42.1)	31 (43.7)	32 (40.0)
**Work experience**, n	228	76	72	80
in years, mean (SD)	25.5 (7.9)	25.8 (7.3)	25.8 (8.6)	24.9 (8.0)

*GP* General Practitioner, *SD* Standard deviation

Since there was a high and statistically significant correlation between age and work experience of participants (Pearson’s *r* = *0.913, p* =  < *0.001*), the variable ‘physician age’ was excluded as previous research found work experience as an important factor that influences the decision-making of antibiotic prescribing [3, 22].

### Descriptive analysis

With 126 (46.8%) physicians, nearly half of participants indicated to have perceived an impact on their decision-making in antibiotic prescribing by partaking in the ARena project (T2). This estimation was homogeneous across all three intervention arms (Chi^2^(2): 0.08, *p* = *0.963*).

The PCN as a contextual factor of the ARena project was assessed as an overall supportive environment with a mean value of 3.9 (SD 0.7, *n* = 224). Regarding the intervention arms I, II and III, group comparisons showed statistically significant differences between the intervention arms regarding the evaluation of the PCN environment (H-Test: 6.50(2), *p* = *0.039*). With a mean value of 4.1 (SD 0.7, *n* = 72), intervention arm II rated their PCN environment as more supportive than intervention arm I (mean: 3.8, SD 0.7, *n* = 73) and intervention arm III (mean: 3.9, SD 0.8, *n* = 79).

Considering the context of the medical practice, most of the respondents worked in catchment areas of less than 100,000 population (52.4%, *n* = 141) and about 32.0% (*n* = 86) in areas of more than 100,000 population. Group comparisons showed statistically significant differences regarding the practice area (Chi^2^(2): 66.25, *p* < *0.001*). There were no significant differences between the intervention arms in other contextual factors (see Additional file 5 Table 1). About 13.0% (*n* = *34*) of the participating physicians indicated to treat less than 1,000 patients and 67.0% (*n* = *180*) to treat more than 1,000 patients per quarter of a year.

While structural conditions (mean: 2.9, SD 1.1) as well as externally defined general conditions (mean 2.7, SD 0.9) of the practice were assessed as a less supportive environment, existing processes and the organisation of the practice as a whole were shown to be considered a supportive environment (mean: 3.3, SD 1.0).

### Bivariate analysis

Bivariate results regarding the relationship between decision-making and contextual factors refer to the number of participants who returned the survey questionnaires at T0 and T2 (*n* = 152). Group comparison showed only marginal differences between participants in the three arms (see Additional file 5 Table 2).

A total of 5 out of 10 predictors showed significant bivariate association with the outcome. 

Table 2 shows statistically significant relationships between the perceived impact of participation in the ARena project on decision-making in antibiotic prescribing and work experience, PCN environment, structural conditions, environment of existing processes, and externally defined general conditions.

**Table 2:** Bivariate analyses of contextual factors and the dependent variable (PIP-ARena)

**Variables**	**N**	**PIP-ARena** *n* = 108 n (%)	**No PIP-ARena** *n* = 43 n (%)	**Correlation**	***P* value**
**Individual characteristics**					
Sex	148	107 (72.3)	41 (27.7)	0.05 ^**a**^	0.578
Female	49	34 (69.4)	15 (30.6)		
Male	99	73 (73.7)	26 (26.3)		
Medical Specialty	149	107 (71.8)	42 (28.2)	0.06 ^**a**^	0.438
GP	89	66 (74.2)	23 (25.8)		
Specialists	60	41 (68.3)	19 (31.7)		
Work experience, mean SD	51	27.0 (8.0)	24.0 (7.0)	0.19 ^**b**^	0.026
**Intervention arm allocation**	151	108 (71.5)	43 (28.5)	0.11 ^**a**^	0.414
I	50	39 (78.0)	11 (22.0)		
II	57	40 (70.2)	17 (29.8)		
III	47	29 (65.9)	15 (34.1)		
**PCN environment,** mean SD	148	4.1 (0.7)	3.7 (0.8)	0.24 ^**b**^	0.003
**General characteristics of medical practice**					
Number of patients per quarter of year	139	103 (74.1)	36 (25.9)	0.04 ^**a**^	0.618
< 1,000 patients	23	18 (78.3)	5 (21.7)		
> 1,000 patients	116	85 (73.3)	31 (26.7)		
Practice area	149	107 (71.8)	42 (28.2)	0.02 ^**a**^	0.841
< 100,000 population	98	71 (72.4)	27 (27.6)		
≥ 100,000 population	51	36 (70.6)	15 (29.4)		
Structural conditions, mean SD	147	3.1 (1.1)	2.5 (1.0)	0.24 ^**b**^	0.004
Environment of existing processes, mean SD	150	3.4 (1.0)	3.0 (1.1)	0.17 ^**b**^	0.004
External defined general conditions, mean SD	149	2.8 (0.9)	2.4 (0.8)	0.18 ^**b**^	0.032

^**a**^CramerV; ^**b**^Eta

*PIP-ARena* perceived impact of participation in the ARena project on the decision-making regarding antibiotic prescribing, *GP* General Practitioner, *PCN* primary care network, *SD* Standard deviation

No statistically significant relationship was observed between the outcome measure and sex, the qualification of physicians, the intervention arm allocation, the number of patients per quarter of year, or the practice catchment area.

### Multivariable analysis

First, multivariable logistic regression models were separately performed for each of the four CICI domains. Results only indicated the same predictive factors as significant as the bivariate analysis (see Additional file 6 for details).

The number of cases limited the inclusion of predictive factors in the multivariable logistic regression model. Thus, only statistically significant predictors in the bivariate analyses were included for further analyses of the influence of the perceived impact of participation in the ARena project on decision-making regarding antibiotic prescribing. First, all factors were examined in bivariate logistic regression models (Table 3).

**Table 3:** Bivariate logistic regression analyses of contextual factors influencing PIP-ARena

Variables	B	SE	Wald	df	*P* value	OR	CI 95%
Experience year	0.05	0.02	4.82	1	0.028	1.05	1.006–1.103
PCN environment	0.72	0.25	8.22	1	0.004	2.06	1.256–3.363
Structural conditions	0.50	0.18	7.74	1	0.005	1.66	1.161–2.371
Environment of existing processes	0.37	0.19	4.08	1	0.043	1.46	1.011–2.094
External defined general conditions	0.45	0.21	4.51	1	0.034	1.57	1.035–2.378

*PIP-ARena* perceived impact of participation in the ARena project on the decision-making regarding antibiotic prescribing, *PCN* primary care network

The bivariate logistic regression analyses showed that both the models as a whole and the individual coefficients of the contextual factors were significant. Nagelkerkes R^2^ was between 0.04 and 0.08. This resulted in a Cohen effect strength of 0.20–0.30.

Practitioners with longer work experience were more likely to perceive an impact on decision-making regarding antibiotic prescribing by participating in the ARena project. Results indicated the more supportive the PCN environment was perceived to be, the more likely participation in the ARena project was perceived to have an impact on decision-making regarding antibiotic prescribing. Higher perceived support from structural conditions, existing processes, and externally defined general conditions increased the relative probability of a perceived impact on decision-making in antibiotic prescribing through participation in the ARena project by 66.0%, 46.0%, and 57.0% respectively.

Subsequently, all factors were included stepwise in a multivariable model. The sample size in the hierarchical logistic regression analysis was *n* = 142, as all variables were present. The model was statistically significant (Chi^2^(5) = 16.13, *p* = *0.006*). Nagelkerkes R^2^ was at 0.15. This resulted in a Cohen effect strength of 0.43 and the Hosmer–Lemeshow test was Chi^2^(8) = 5.71, *p* = *0.680*.

While all factors showed a significant influence in the univariate regression models, only work experience OR 1.05 (95% CI 1.001–1.104) continuously showed a significant influence in the multivariable model (Table 4).

**Table 4:** Multivariable logistic regression analysis of influencing factors on PIP-ARena (*n* = 142)

Variables	B	SE	Wald	df	*P* value	OR	CI 95%
Experience year	0.05	0.03	3.96	1	0.046	1.05	1.001–1.104
PCN environment	0.45	0.29	2.41	1	0.121	1.57	0.889–2.763
Structural conditions	0.14	0.29	0.24	1	0.626	1.15	0.648–2.054
Environment of existing processes	0.14	0.29	0.25	1	0.618	1.16	0.656–2.032
External defined general conditions	0.20	0.29	0.50	1	0.481	1.22	0.699–2.136
Constant	-3.43	1.26	7.46	1	0.006	0.03	

*PIP-ARena* perceived impact of participation in the ARena project on the decision-making regarding antibiotic prescribing, *PCN* primary care network

In order to take the nested data structure into account, the ICC was calculated and identified at 0.22. The effect sizes in the MLA marginally differed from the results in the multivariable logistic regression analysis and were statistically unremarkable. An additional table file shows this in more detail (see Additional file 7). Due to this, it was decided to present the results of the multivariable logistic regression in detail for better comprehensibility. However, due to the small number of clusters and the small number of cases within the clusters, the risk of bias in the estimations of effects must be considered.

## Discussion

### Main results

Nearly half of the participating primary care physicians reported to have perceived an impact on their decision-making regarding antibiotic prescribing which was attributed to participation in the ARena project. With increasing work experience, the likelihood that participation in the ARena project was perceived as influential on antibiotic prescribing decision-making increased. Other factors, such as the PCN environment, structural conditions, environment of existing processes, or externally defined general conditions showed a significant positive influence in the bivariate logistic regression analysis. None of these effects were confirmed in the multivariable logistic regression model. This might be due to the small sample size and the number of predictive factors and have resulted in a lack of statistical power to detect effects. Other contextual factors such as practice size or practice catchment area did not have a statistically significant influence. The intervention arm affiliation did not have a statistically significant influence on decision-making on antibiotic prescribing due to participation in the ARena project either.

### Comparison to previous research

Prior research investigated the influence of work experience on antibiotic prescribing decisions with heterogeneous findings. In two international interview studies [23, 24], physicians reported a perceived influence of work experience on their antibiotic prescribing decision. Furthermore, a Canadian study that evaluated linked administrative health care data found that primary care physicians who were mid-career or more advanced in their career were more likely to prescribe antibiotics than physicians with less work experience [25]. However, other results indicated no influence of work experience on antibiotic prescribing decisions [26]. These different findings indicate closer examination in further efforts.

According to the TPB, the significant positive influence of work experience in this study could be interpreted asa well perceived behavioral control and attitude towards a sustainable use of antibiotics, presumably based on many years of professional experience in antibiotic prescribing and accrued knowledge about antibiotics and AMR. This might indicate that with increasing work experience and thus accumulated reference points, physicians' confidence in the ability to perform the desired behavior might rise [16]. This assumption is supported by prior research that reported a significant lack of knowledge about antibiotics and AMR particularly in the group of physicians with little work experience and potentially paired with uncertainty in decision-making regarding antibiotic prescriptions [27]. On the other hand, research also shows that younger physicians might more likely be guideline-oriented than more experienced physicians [28], who may be less current with evidence and guideline-based care than younger physicians [27]. The effect measured in this study indicates that more experienced physicians perceive participation in a program that uses knowledge-transmitting intervention measures to have an impact on their prescribing decisions. This assumption should be further investigated in future research.

The detected effect could also support the assumption of the project's implementation strategy, which specifically targeted knowledge deficits regarding antibiotics and AMR as well as physicians’ awareness regarding their own prescribing routines. Confirming this assumption, the outcome evaluation of the ARena project observed a significant reduction of antibiotic prescribing in the intervention arms in a pre-post comparison and compared to matched standard care, suggesting that the implementation program had impact on professional practice [14, 15].

Findings of this study suggest that PCNs were a supportive environment in the ARena project. In the bivariate regression, a positive influence on decision-making in antibiotic prescribing was shown. A qualitative study within the process evaluation of ARena pointed in the same direction [21]: Physicians described PCNs as supportive with regard to exchange (beliefs, ideas and experiences), management, and the implementation of new routines in practice, which can influence decision-making on antibiotic prescribing [21, 29]. Research further showed that PCNs can act as effective drivers of innovation and quality improvement, especially when communication and support functioned well within the networks [21, 30, 31]. Being part of a PCN can thus support the adoption of specific behaviors and beliefs [13, 21]. With regards to the TPB, PCNs as a contextual factor might have had a strong influence on both social norms and attitudes towards the targeted behavior through social interactions and relationships. The analysis showed that physicians in arm II rated the PCN environment more supportive than physicians in arm I and/or III. This could be due to the implementation strategy since in arm II, medical assistants were to be strongly involved in the intervention’s awareness creating efforts to support physicians and patients alike. This may have been seen as an additional supportive factor and might have had a reinforcing effect on the perception of PCNs as a supportive environment in arm II.

When interpreting results, it should be considered that PCN member physicians might show stronger tendencies to innovation and development than physicians who are not part of a PCN [13, 21]. The data analyzed in this study were generated in the process evaluation carried out alongside ARena. Therefore, comparisons with standard care (no intervention) could not be performed. Participants might have given socially desirable answers on the survey questionnaires. Potential biases due to the clustered structures in PCNs were accounted for by the MLA where effect sizes marginally differed from the results in the multivariable logistic regression analysis and were statistically unremarkable. However, for further interpretation and discussion of the results, it should be considered that there was a slight cluster effect.

Few reference studies were found which also address general conditions of medical practices. For example, some studies showed that communication and the organisational model have an influence on antibiotic prescribing [26, 32, 33]. However, the results of the bivariate regression in this study show that specific contextual factors such as structural conditions, environment of existing processes, or externally defined general conditions had an influence on participation in the project and influenced decision-making on antibiotic prescribing. Due to the mean score variables used in this analysis, a differentiated consideration of the domains is not possible. Nevertheless, the results indicate that such general conditions may have an effect regarding antibiotic prescribing decisions and might be a facilitating or inhibiting factor in an implementation process. As these factors are highly individualized at physician level, practice level, or health system level, it is difficult to compare different study samples at all levels or generalize results. Further research is needed regarding such contextual factors to analyze their impact in detail. In the TPB, Ajzen [16] described the influence of similar conditions such as legal requirements, availability of resources, or cooperations on so-called actual behavioral control. Behavioral control and motivation to behave can both significantly influence the intention and, consequently, decision-making [16].

While one study identified a significant influence of practice area on antibiotic prescribing [22], this study found no influence. Furthermore, a study showed that physicians who treated a high number of patients were more likely to prescribe antibiotics [25]. This cannot be confirmed by this study either. In total, it can be seen that general conditions of the medical practice, such as the practice size or practice area, as well as specific conditions as described above, were hardly considered or mentioned in research before [34].

No effects could be attributed to the affiliation to the intervention arm. This could be due to the fact that all groups largely received the same intervention components and that the effects of the additional intervention components in arms II and III, in addition to a small number of cases, were not statistically strong enough. Also, in this analysis, only the assignment to the intervention arm was used as a predictor. More detailed analyses on the uptake of the individual intervention components and their possible influence on physicians’ perception of antibiotic prescribing are therefore needed.

Overall, previous studies which explored contextual influencing factors were mainly qualitative. Statistical correlations in the specific context of decision-making regarding the prescription of antibiotics were only examined to a limited extent. This study therefore contributes to the identification of statistical correlations regarding this topic as a first of its kind. As described in the introduction, the description and differentiation of the concept of context is complex. In the CICI, context is differentiated into seven domains [10]. In this study, the predictive factors were classified into all contextual domains related to individual factors and the intervention arm allocation in order to examine their influence and relationships in terms of the CICI. Due to the number of cases, only a few numbers of factors could be analyzed. The large number of contextual factors given the small number of cases can be best considered indicative of relationships and influences only. However, investigating to which extend they jointly influenced decision-making in antibiotic prescribing was limited. Large-scale studies are needed to sufficiently investigate a diverse environment around physicians’ practices in intervention studies. By doing so, effects between context and CMCI could be identified and contextual factors in the sense of the CICI could be covered as broadly as possible.

### Strengths and limitations

Several strengths and limitations must be considered when interpreting the results of this study. One strength of this study was the application of the STROBE-Statement which granted methods and results to be transparent and comprehensible.

The applied questionnaire was not validated but theory-based in conceptualization. However, it can be assumed that all questions were well understood since the number of missing values was small and they were completely at random. Another strength of the study was the high response rate. Nevertheless, the number of cases was statistically too small to conduct a comprehensive analysis of all possible contextual factors identified in theory [17]. The inclusion of predictive variables was thus limited in multivariable regression analysis and only significant factors of the bivariate regression analysis were included. Given the relatively small sample size and a potential lack of statistical power, the number of included factors in the multivariable regression analysis might have reduced the degrees of freedom so that effects were not detected. The study focused on the physicians’ perceived impact on decision-making regarding antibiotic prescribing through their participation in the ARena project. This is a self-reported impact, which may contain an over- as well as an underestimation of individual performance. In addition, it was not possible to link the results of the process evaluation to the actual prescribing rate from the intervention study. Therefore, no statement can be made about the influence of the contextual factors on actual prescribing behavior.

Finally, the generalizability of the results of this study is limited. On the one hand, the participants’ age and work experience might limit the transferability of results to younger primary care physicians. On the other hand, the study setting in PCNs must be considered when transferring results. The nested data cannot exclude the possibility that socially desirable responses were given. A potential selection bias might be present in participants' pre-existing motivation to reduce antibiotic use. Furthermore, the contextual factors used in this study are specific to the German health care system. Thus, the results cannot simply be transferred to practitioners who do not participate in a PCN as well as to non-German health systems.

## Conclusion

This study showed that primary care physicians perceived an influence on their decision-making on antibiotic prescribing through participation in the ARena project. In particular, longer work experience was shown to be a significant influential factor. This might indicate that knowledge transfer interventions promoting rational prescribing of antibiotics might be successful when particularly more experienced physicians are targeted.

Contextual factors such as PCN environment, structural conditions, environment of existing processes in the medical practice, and externally defined general conditions also showed to have an influence. An effective synergy of contextual factors regarding decision-making was not observed. Further comparable analyses with larger case numbers could comprehensively examine contextual factors on antibiotic prescribing decision-making. This study contributes to the understanding of contextual factors, their influence on prescribing decision-making, and their role in an implementation strategy.

## Supplementary Information

**Additional file 1. **Study design. 1. ARena study design, 2. Implementation strategy and intervention components, 3. Process evaluation design.

**Additional file 2.** Translated Overview of all relevant questions. 1. Relevant questions from Questionnaire T0, 2. Relevant question from Questionnaire T2.

**Additional file 3.** Overview and Reliability of the mean score variables. a. PCN environment, b. Structural conditions, c. Environment of existing processes, d. External defined general conditions.

**Additional file 4.** Missing value analysis.

**Additional file 5.** Results intervention arm comparison. 1. Results intervention arm comparison (Sample T0), 2. Results intervention arm comparison (Sample T0/T2).

**Additional file 6.** Results multivariable regression analysis according to the four CICI domains. 1. Individual characteristics, 2. Intervention arm allocation, 3. PCN environment, 4. General characteristics of the medical practice.

**Additional file 7.** Results multilevel analysis.

## Data Availability

The datasets used and analysed during the current study are available from the corresponding author on reasonable request. An overview of all relevant survey items for this analysis can be found in Additional file 2.

## Abbreviations

AMR: Antimicrobial Resistance
ARena: Sustainable reduction of antibiotic-induced antimicrobial resistance
CI: Confidence Interval
CICI: The Context and Implementation of Complex Interventions
CMCI: Complex Multiple Component Interventions
DDD: Defined Daily Dose
GP: General Practitioner
ICC: Intra-Cluster Correlation Coefficient
MA: Medical Assistant
MLA: Multilevel Analysis
OR: Odds Ratio
PCN: Primary Care Network
SD: Standard Deviation
TPB: Theory of Planned Behaviour
