# Supplementary material for: Contextual factors influencing physicians’ perception of antibiotic prescribing in primary care in Germany — a prospective observational study
Source: BMC Health Serv Res. 2022 Mar 12;22:331. doi: 10.1186/s12913-022-07701-3 (PMC8917632; doi:10.1186/s12913-022-07701-3)
Supplement: Supplementary file 1 — Additional file 1. Study design. 1. ARena study design, 2. Implementation strategy and intervention components, 3. Process evaluation design. [file 12913_2022_7701_MOESM1_ESM.pdf]

Additional file 1

Study design

1. ARena study design

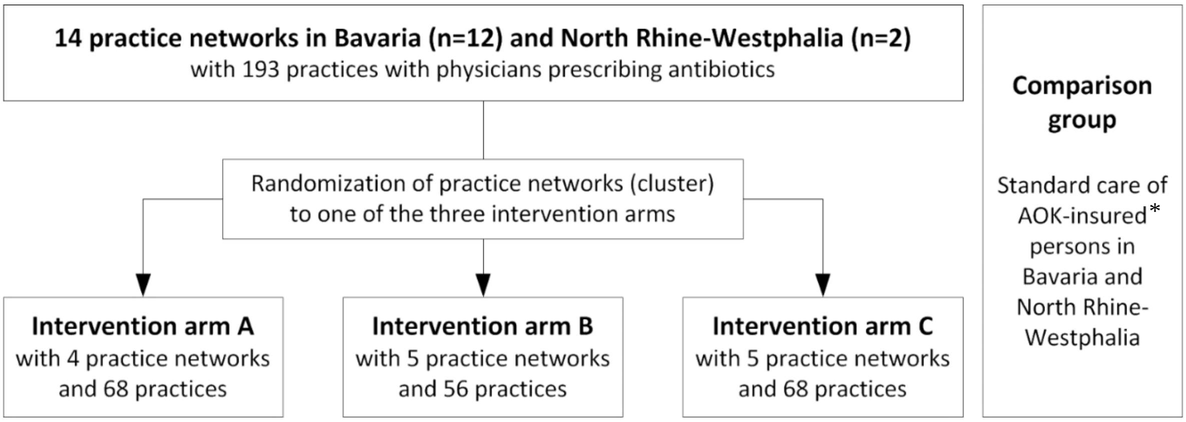

*AOK is a large statutory health insurance provider in Germany

Figure 1: *ARena study design with three intervention arms and comparison group*

Published in: Kamradt M, Kaufmann-Kolle P, Andres E, Brand T, Klingenberg A, Glassen K et al. Sustainable reduction of antibiotic-induced antimicrobial resistance (ARena) in German ambulatory care: study protocol of a cluster randomised trial. Implement Sci 2018; 13(1):23.

2. Implementation strategy and intervention components

The three intervention arms in ARena focused on

- guideline-oriented care, information and education (standard set of intervention components in intervention arms I, II, and III)
- involvement of practice team (intervention arm II only)
- interdisciplinary, cross-sectoral cooperation, software-based decision support (intervention arm III only)

Standard set of implementation components (arms I, II, and III):

Targeting physicians: E-learning on communication with patients, quality circles with data-based feedback, performance-based additional bonus compensation;

Targeting patients: educational information material (poster, leaflets)

Public information campaigns using print, TV, radio, and social media

Add-on in arm II only:

Targeting practice team/MAs: E-learning on communication with patients, quality circles with data-based feedback; tablet pcs to provide educational content to patients in waiting areas

Add-on in arm III only:

Targeting physicians: Interdisciplinary, cross sectoral quality circles, software-based decision support integrated into administrative practice software

3. Process evaluation design

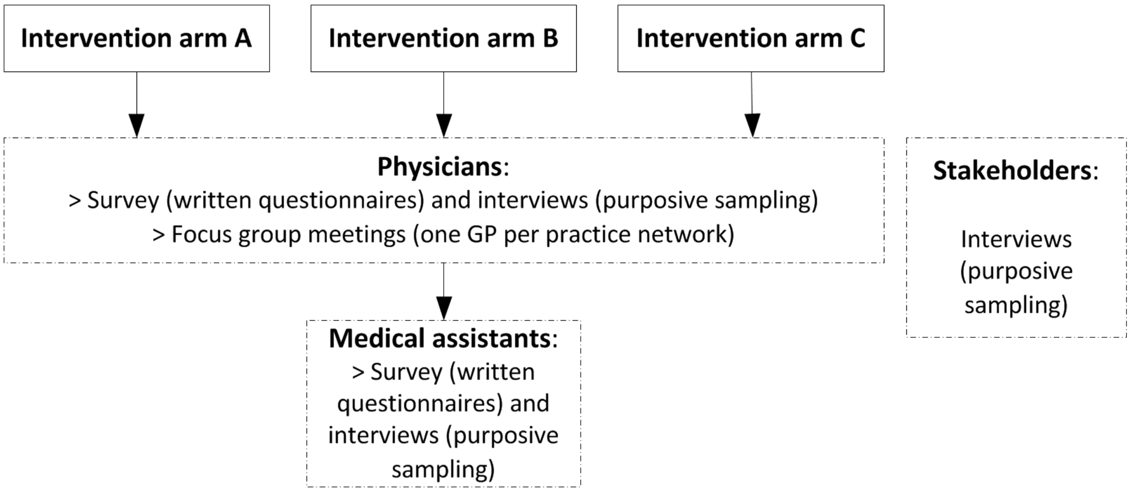

Figure 2: *Process evaluation design of the ARena study*

Published in: Kamradt M, Kaufmann-Kolle P, Andres E, Brand T, Klingenberg A, Glassen K et al. Sustainable reduction of antibiotic-induced antimicrobial resistance (ARena) in German ambulatory care: study protocol of a cluster randomised trial. Implement Sci 2018; 13(1):23.
