# Supplementary material for: Contextual factors influencing physicians’ perception of antibiotic prescribing in primary care in Germany — a prospective observational study
Source: BMC Health Serv Res. 2022 Mar 12;22:331. doi: 10.1186/s12913-022-07701-3 (PMC8917632; doi:10.1186/s12913-022-07701-3)
Supplement: Supplementary file 2 — Additional file 2. Translated Overview of all relevant questions. 1. Relevant questions from Questionnaire T0, 2. Relevant question from Questionnaire T2. [file 12913_2022_7701_MOESM2_ESM.pdf]

Additional file 2

Translated Overview of all relevant questions

1. **Relevant questions from Questionnaire T0**

**Contextual factors**					
**Structural conditions (team, rooms)**	**Strongly disagree**	**Disagree**	**Neutral**	**Agree**	**Strongly agree**
… motivate me to treat patients increasingly guideline-based	☐	☐	☐	☐	☐
… support me in taking shared therapy decisions with the patients	☐	☐	☐	☐	☐
… support me in handling patient expectations regarding the prescription of antibiotics	☐	☐	☐	☐	☐
… are helpful when implementing new routines in the practice	☐	☐	☐	☐	☐
… impact the amount of time available to me per patient	☐	☐	☐	☐	☐
… impact my decision to prescribe antibiotics	☐	☐	☐	☐	☐

**Existing processes and organizing processes in the practice …**	**Strongly disagree**	**Disagree**	**Neutral**	**Agree**	**Strongly agree**
… motivate guideline-oriented patient care	☐	☐	☐	☐	☐
… support me in taking a shared the therapy decision with the patients	☐	☐	☐	☐	☐
… supports me in managing patient	☐	☐	☐	☐	☐

expectations regarding the prescription of antibiotics					
… are helpful with implementing new routines in the practice	☐	☐	☐	☐	☐
… impact the amount of time available to me per patient	☐	☐	☐	☐	☐
… impact my decision to prescribe antibiotics	☐	☐	☐	☐	☐

**External defined general conditions…**	**Strongly disagree**	**Disagree**	**Neutral**	**Agree**	**Strongly agree**
... motivate guideline-oriented patient care	☐	☐	☐	☐	☐
... support me in taking a shared the therapy decision with the patients	☐	☐	☐	☐	☐
... supports me in managing patient expectations regarding the prescription of antibiotics	☐	☐	☐	☐	☐
... are helpful with implementing new routines in the practice	☐	☐	☐	☐	☐
... impact the amount of time available to me per patient	☐	☐	☐	☐	☐
... impact my decision to prescribe antibiotics	☐	☐	☐	☐	☐

**Participating in the network ...**	**Strongly disagree**	**Disagree**	**Neutral**	**Agree**	**Strongly agree**
… motivates guideline-oriented patient care	☐	☐	☐	☐	☐

... supports shared-decision making	☐	☐	☐	☐	☐
... supports managing patient expectations regarding the prescription on antibiotics	☐	☐	☐	☐	☐
... supports implementing new routines	☐	☐	☐	☐	☐
... has an impact on my antibiotic prescribing decisions	☐	☐	☐	☐	☐

**In my primary care network**	**Strongly disagree**	**Disagree**	**Neutral**	**Agree**	**Strongly agree**
… antibiotics therapy is discussed	☐	☐	☐	☐	☐
… peer exchange about guideline-oriented antibiotics therapy is offered	☐	☐	☐	☐	☐
… exchange about antibiotic prescribing routines for non-complicated infections is possible	☐	☐	☐	☐	☐
… there are conventions about antibiotics for non-complicated infections	☐	☐	☐	☐	☐
... training on guideline-oriented antibiotics therapy is offered	☐	☐	☐	☐	☐
… I participated in training on guideline-oriented antibiotics therapy	☐	☐	☐	☐	☐

General information about person and medical practice

Your year of birth?	|__|__|__|__| (Please enter a year here)
Sex?	☐ female ☐ male
Your medical specialty?	____________________<br>____________________
How many professional years of experience as a physician do you have?	ca. |__|__| years
Your employment status?	☐ full-time ☐ self-employed<br>☐ part-time ☐ employed
What is the size of the community where your practice is located?	☐ < 5.000 inhabitants<br>☐ 5.000 to under 20.000 inhabitants<br>☐ 20.000 to under 100.000<br>☐ 100.000 to under 500.000 inhabitants<br>☐ > 500.000 inhabitants
In which type pf practice are you working?	☐ single practice ☐ shared rooms<br>☐ group practice ☐ medical center
How many patients do you see per quarter of year on average?	☐ < 500 ☐ 1.001-1.500<br>☐ 500-1.000 ☐ > 1.500

2. Relevant question from Questionnaire T2

**Prescription of medication**					
My decision to prescribe antibiotics is influenced by...	**Strongly disagree**	**Disagree**	**Neutral**	**Agree**	**Strongly agree**
... my participation in the ARena project	☐	☐	☐	☐	☐
