# Supplementary material for: Contextual factors influencing physicians’ perception of antibiotic prescribing in primary care in Germany — a prospective observational study
Source: BMC Health Serv Res. 2022 Mar 12;22:331. doi: 10.1186/s12913-022-07701-3 (PMC8917632; doi:10.1186/s12913-022-07701-3)
Supplement: Supplementary file 3 — Additional file 3. Overview and Reliability of the mean score variables. a. PCN environment, b. Structural conditions, c. Environment of existing processes, d. External defined general conditions. [file 12913_2022_7701_MOESM3_ESM.pdf]

Additional file 3

Overview and Reliability of the mean score variables

a. PCN environment

Reliability Statistics

Cronbach's Alpha	N of Items	N
0.898	11	224

Item-Total Statistics

Participating in the network ...	Scale Mean if Item Deleted	Scale Variance if Item Deleted	Corrected Item-Toal Correlation	Cronbach's Alpha if Item Deleted
… motivates guideline-oriented patient care	39.38	52.51	0.73	0.882
... supports shared-decision making	39.64	51.63	0.71	0.883
... supports managing patient expectations regarding the prescription on antibiotics	39.63	52.02	0.71	0.884
... supports implementing new routines	39.31	53.08	0.69	0.885
... has an impact on my antibiotic prescribing decisions	40.13	52.30	0.58	0.894
**In my primary care network**				
… antibiotics therapy is discussed	38.74	58.99	0.48	0.896
… peer exchange about guideline-oriented antibiotics therapy is offered	39.07	55.54	0.65	0.888
… exchange about antibiotic prescribing routines for non-complicated infections is possible	39.24	54.52	0.63	0.888
… there are conventions about antibiotics for non-complicated infections	39.38	53.20	0.65	0.887
... training on guideline-oriented antibiotics therapy is offered	38.76	57.55	054	0.893
… I participated in training on guideline-oriented antibiotics therapy	38.81	56.70	0.57	0.892

b. Structural conditions

Reliability Statistics

Cronbach's Alpha	N of Items	N
0.921	6	221

Item-Total Statistics

Structural conditions (teams, rooms)	Scale Mean if Item Deleted	Scale Variance if Item Deleted	Corrected Item-Toal Correlation	Cronbach's Alpha if Item Deleted
… motivate me to treat patients increasingly guideline-based	14.61	29.49	0.82	0.901
... support me in taking shared therapy decisions with the patients	14.48	29.25	0.87	0.893
... support me in handling patient expectations regarding the prescription of antibiotics	14.53	29.33	0.89	0.891
... are helpful when implementing new routines in the practice	14.12	31.68	0.75	0.910
... impact the amount of time available to me per patient	14.18	32.82	0.61	0.929
... impact my decision to prescribe antibiotics	15.06	31.24	0.72	0.915

c. Environment of existing processes

Reliability Statistics

Cronbach's Alpha	N of Items	N
0.907	6	224

Item-Total Statistics

Existing processes and organizing processes in the practice ...	Scale Mean if Item Deleted	Scale Variance if Item Deleted	Corrected Item-Toal Correlation	Cronbach's Alpha if Item Deleted
... motivate guideline-oriented patient care	16.43	23.35	0.82	0.879
... support me in taking a shared the therapy decision with the patients	16.29	23.73	0.85	0.875
... supports me in managing patient expectations regarding the prescription of antibiotics	16.42	23.44	0.86	0.873
... are helpful with implementing new routines in the practice	16.06	25.39	0.72	0.894
... impact the amount of time available to me per patient	16.16	25.78	0.56	0.917
... impact my decision to prescribe antibiotics	17.04	23.67	0.69	0.900

d. External defined general conditions

Reliability Statistics

Cronbach's Alpha	N of Items	N
0.890	6	224

Item-Total Statistics

External defined general conditions…	Scale Mean if Item Deleted	Scale Variance if Item Deleted	Corrected Item-Toal Correlation	Cronbach's Alpha if Item Deleted
... motivate guideline-oriented patient care	13.16	22.41	0.74	0.865
... support me in taking a shared the therapy decision with the patients	13.35	21.30	0.83	0.851
... supports me in managing patient	13.4	21.29	0.84	0.849

expectations regarding the prescription of antibiotics				
... are helpful with implementing new routines in the practice	13.34	22.24	0.76	0.863
... impact the amount of time available to me per patient	13.22	25.21	0.35	0.930
... impact my decision to prescribe antibiotics	13.62	21.47	0.80	0.857
