# Supplementary material for: Contextual factors influencing physicians’ perception of antibiotic prescribing in primary care in Germany — a prospective observational study
Source: BMC Health Serv Res. 2022 Mar 12;22:331. doi: 10.1186/s12913-022-07701-3 (PMC8917632; doi:10.1186/s12913-022-07701-3)
Supplement: Supplementary file 4 — Additional file 4. Missing value analysis. [file 12913_2022_7701_MOESM4_ESM.pdf]

Additional file 4

Missing value analysis

Univariate Statistics

	N	Mean	SD	Missing		No. Of Extremes[a]	
				Count	Percent	Low	High
work_experience_year_T0	151	25.91	7.99	1	0.7	0	1
age_T0	147	54.81	7.50	5	3.3	3	0
sex_T0	149	0.67	0.47	3	2.0	0	0
medical_speciality_T0	150	1.89	1.63	2	1.3	0	15
patient_quantity_T0	140			12	7.9		
practice_area_T0	150			2	1.3		
**Structural conditions**							
structure_guide_T0<br>… motivate me to treat patients increasingly guideline-based	149			3	2.0		
structure_involve_T0<br>… support me in taking shared therapy decisions with the patients	151			1	0.7		
structure_expect_T0<br>… support me in handling patient expectations regarding the prescription of antibiotics	151			1	0.7		
structure_change_T0<br>… are helpful when implementing new routines in the practice	150			2	1.3		
structure_time_T0<br>… impact the amount of time available to me per patient	151			1	0.7		
structure_impact_T0<br>… impact my decision to prescribe antibiotics	151			1	0.7		
**Existing processes and organizing processes in the practice …**							
orga_guide_T0<br>… motivate guideline-oriented patient care	151			1	0.7		

orga_involve_T0<br>… support me in taking a shared the therapy decision with the patients	151			1	0.7			
orga_expect_T0<br>… supports me in managing patient expectations regarding the prescription of antibiotics	151			1	0.7			
orga_change_T0<br>… are helpful with implementing new routines in the practice	151			1	0.7			
orga_time_T0<br>… impact the amount of time available to me per patient	151			1	0.7			
orga_impact_T0<br>… impact my decision to prescribe antibiotics	151			1	0.7			
**External defined general conditions …**								
regulation_guide_T0<br>... motivate guideline-oriented patient care	151			1	0.7			
regulation_decision_T0<br>... support me in taking a shared the therapy decision with the patients	152			0	0			
regulation_expect_T0<br>... supports me in managing patient expectations regarding the prescription of antibiotics	152			0	0			
regualtion_change_T0<br>... are helpful with implementing new routines in the practice	152			0	0			
regulation_time_T0<br>... impact the amount of time available to me per patient	152			0	0			
regulation_impact_T0<br>... impact my decision to prescribe antibiotics	151			1	0.7			

**Participating in the network ...**							
PCN_guide_T0<br>… motivates guideline-oriented patient care	152			0	0		
PCN_involve_T0<br>... supports shared-decision making	152			0	0		
PCN_expect_T0<br>... supports managing patient expectations regarding the prescription on antibiotics	152			0	0		
PCN_change_T0<br>... supports implementing new routines	152			0	0		
PCN_impact_T0<br>... has an impact on my antibiotic prescribing decisions	151			1	0.7		
**In my primary care network**							
PCN_subject_T0<br>… antibiotics therapy is discussed	152			0	0		
PCN_exchange_gen_T0<br>… peer exchange about guideline-oriented antibiotics therapy is offered	152			0	0		
PCN_exchange_specif_T0<br>… exchange about antibiotic prescribing routines for non-complicated infections is possible	152			0	0		
PCN_agreement_T0<br>… there are conventions about antibiotics for non-complicated infections	152			0	0		
PCN_training_T0<br>... training on guideline-oriented antibiotics therapy is offered	151			1	0.7		
PCN_training_used_T0<br>… I participated in training on guideline-oriented antibiotics therapy	151			1	0.7		

[a] Number of cases outside the range (Q1 – 1.5*IQR, Q3 + 1.5*IQR).

EM-Correlation[a]

	work_experience_year_T0	age_T0	sex_T0	medical_speciality_T0
work_experience_year_T0	1			
age_T0	0.922	1		
sex_T0	0.12	0.112	1	
medical_speciality_T0	0.011	0	0.209	1

[a] Little's MCAR test: Chi-Square = 13.741, DF = 10, Sig. = .185
