# Supplementary material for: Contextual factors influencing physicians’ perception of antibiotic prescribing in primary care in Germany — a prospective observational study
Source: BMC Health Serv Res. 2022 Mar 12;22:331. doi: 10.1186/s12913-022-07701-3 (PMC8917632; doi:10.1186/s12913-022-07701-3)
Supplement: Supplementary file 5 — Additional file 5. Results intervention arm comparison. 1. Results intervention arm comparison (Sample T0), 2. Results intervention arm comparison (Sample T0/T2). [file 12913_2022_7701_MOESM5_ESM.pdf]

Additional file 5

1 Results intervention arm comparison (Sample T0)

Variables	Total N (%)	Intervention arm I n (%)	Intervention arm II n (%)	Intervention arm III n (%)	Test	P value
**PIP-ARena**	183	68	66	49		
PIP-ARena	126 (68.9)	47 (69.1)	46 (69.7)	33 (67.3)	0.076 [a]	0.963
No PIP-ARena	57 (31.1)	21 (30.9)	20 (30.3)	16 (32.7)		
**Individual characteristics**						
**Sex**	224	73	73	78	5.245 [a]	0.073
Female	76 (33.9)	18 (24.7)	25 (34.2)	33 (42.3)		
Male	148 (66.1)	55 (75.3)	48 (65.8)	45 (57.7)		
**Work experience**	228	76	72	80	1.320 [b]	0.517
in years, mean (SD)	25.5 (7.9)	25.8 (7.3)	25.8 (8.6)	24.9 (8.0)		
**Medical speciality**	227	76	71	80	0.210 [a]	0.900
GP	132 (58.1)	44 (57.9)	40 (56.3)	48 (60.0)		
Specialists	95 (41.9)	32 (42.1)	31 (43.7)	32 (40.0)		
**PCN environment**	224	73	72	79	6.499 [b]	0.039
Mean (SD)	3.9 (0.7)	3.8 (0.7)	4.1 (0.7)	3.9 (0.8)		
**General characteristics of medical practice**						
**Number of patients per quarter of year**	214	74	61	79	0.297 [a]	0.862
< 1,000 patients	34 (13.0)	11 (14.9)	11 (18.0)	12 (15.9)		
> 1,000 patients	180 (67)	63 (85.1)	50 (82.0)	67 (84.8)		
**Practice area**	227	76	73	78	66.251 [a]	< 0.001
< 100,000 population	141 (52.4)	68 (89.5)	19 (26.0)	54 (69.2)		
> 100,000 population	86 (32.0)	8 (10.5)	54 (74.0)	24 (30.8)		
**Structural conditions**	221	72	71	78	0.360 [b]	0.835
Mean (SD)	2.9 (1.1)	2.8 (1.2)	3.0 (1.1)	2.9 (1.0)		
**Environment of existing processes**	224	74	72	78	0.510 [b]	0.775
Mean (SD)	3.3 (1.0)	3.2 (1.0)	3.4 (1.0)	3.3 (0.9)		
**External defined general conditions**	224	74	73	77	0.383 [b]	0.826
Mean SD	2.7 (0.9)	2.7 (1.0)	2.7 (1.0)	2.7 (0.9)		

[a]= Chi^2 ; [b]= Kruskal-Wallis Test

PIP-ARena = perceived impact of participation in the ARena project on the decision-making regarding antibiotic prescribing; GP = General Practitioner; PCN = primary care network; SD = Standard deviation

2 Results intervention arm comparison (Sample T0/T2)

Variables	Total N (%)	Intervention arm I n (%)	Intervention arm II n (%)	Intervention arm III n (%)	Test	P value
**PIP-ARena**	151	50	57	44		
PIP-ARena	108 (71.5)	39 (78.0)	40 (70.2)	29 (65.9)	1.762 [a]	0.414
No PIP-ARena	43 (28.5)	11 (22.0)	17 (29.8)	15 (34.1)		
**Individual characteristics**						
**Sex**	149	49	58	42	2.879 [a]	0.237
Female	49 (32.9)	13 (26.5)	18 (31.0)	18 (42.9)		
Male	100 (67.1)	36 (73.5)	40 (69.0)	24 (57.1)		
**Work experience**	151	50	57	44	0.368 [b]	0.832
in years, mean (SD)	25.9 (8.0)	25.3 (7.0)	26.2 (8.6)	26.3 (8.4)		
**Medical speciality**	150	50	56	44	0.588 [a]	0.745
GP	90 (60.0)	28 (56.0)	34 (60.7)	28 (63.6)		
Specialists	60 (40.0)	22 (44.0)	22 (39.3)	16 (36.4)		
**PCN environment**	149	48	57	44	6.601 [b]	0.037
Mean (SD)	4.0 (0.7)	3.8 (0.7)	4.1 (0.7)	3.9 (0.8)		
**General characteristics of medical practice**						
**Number of patients per quarter of year**	140	49	48	43	1.034 [a]	0.596
< 1,000 patients	23 (16.4)	7 (14.3)	10 (20.8)	6 (14.0)		
> 1,000 patients	117 (83.6)	42 (85.7)	38 (79.2)	37 (86.0)		
**Practice area**	150	50	58	42	48.987 [a]	< 0.001
< 100,000 population	99 (66.0)	47 (94.0)	19 (32.8)	33 (78.6)		
> 100,000 population	51 (34.0)	3 (6.0)	39 (69.2)	9 (21.4)		
**Structural conditions**	148	48	56	44	0.431 [b]	0.806
Mean (SD)	2.9 (1.1)	2.8 (1.2)	3.0 (1.2)	2.9 (0.9)		
**Environment of existing processes**	151	50	57	44	0.401 [b]	0.818
Mean (SD)	3.3 (1.0)	3.3 (1.0)	3.3 (1.0)	3.2 (0.9)		
**External defined general conditions**	150	49	58	43	0.210 [b]	0.900
Mean SD	2.7 (0.9)	2.6 (1.0)	2.7 (0.9)	2.7 (0.8)		

[a]= Chi^2 ; [b]= Kruskal-Wallis Test

PIP-ARena = perceived impact of participation in the ARena project on the decision-making regarding antibiotic prescribing; GP = General Practitioner; PCN = primary care network; SD = Standard deviation
