# Supplementary material for: Contextual factors influencing physicians’ perception of antibiotic prescribing in primary care in Germany — a prospective observational study
Source: BMC Health Serv Res. 2022 Mar 12;22:331. doi: 10.1186/s12913-022-07701-3 (PMC8917632; doi:10.1186/s12913-022-07701-3)
Supplement: Supplementary file 6 — Additional file 6. Results multivariable regression analysis according to the four CICI domains. 1. Individual characteristics, 2. Intervention arm allocation, 3. PCN environment, 4. General characteristics of the medical practice. [file 12913_2022_7701_MOESM6_ESM.pdf]

Additional file 6

Results multivariable logistic regression analysis according to the four CICI domains

1. Individual characteristics

Variables	B	SE	Wald	df	P value	OR	CI 95%
Sex, male	0.40	0.44	0.82	1	0.365	1.49	0.628–3.544
Experience year	0.05	0.03	3.75	1	0.053	1.06	0.999–1.120
Med. Speciality, GP	-0.41	0.43	0.92	1	0.339	0.66	0.285–1.541
Self employed	1.23	1.31	0.89	1	0.346	3.42	0.265–44.191
Constant	-1.72	1.32	1.69	1	0.193	0.18	

Dependent Variable: perceived impact of participation in the ARena project on decision-making on antibiotic prescribing; GP = general practitioner

2. Intervention arm allocation

Variables	B	SE	Wald	df	P value	OR	CI 95%
Arm I			1.74	2	0.42		
Arm II	-0.41	0.49	0.84	1	0.360	0.66	0.276–1.596
Arm III	0.61	0.47	1.69	1	0.194	0.55	0.219–1.361
Constant	1.27	0.34	13.74	1	0.000	3.55	

Dependent Variable: perceived impact of participation in the ARena project on decision-making on antibiotic prescribing

3. PCN environment

Variables	B	SE	Wald	df	P value	OR	CI 95%
PCN environment	0.72	0,25	8.22	1	0.004	2.06	1.256-3.363
Constant	-1.84	0.98	3.54	1	0.060	3.55	

Dependent Variable: perceived impact of participation in the ARena project on decision-making on antibiotic prescribing; PCN = primary care network

4. General characteristics of the medical practice

Variables	B	SE	Wald	df	P value	OR	CI 95%
Number of patients per quarter of year (> 1,000 patients)	-0.05	0.57	0.01	1	0.932	0.95	0.311-2.915
Practice area (≥ 100,000 population)	-0.37	0.43	0.76	1	0.384	0.69	0.297-1.597
Structural conditions	0.07	0.31	0.05	1	0.817	1.07	0.586-1.972
Environment of existing processes	0.32	0.30	1.10	1	0.295	1.37	0.760-2.477
External defined general conditions	0.20	0.28	0.52	1	0.473	1.22	0.711-2.088
Constant	-0.53	0.92	0.33	1	0.568	0.59	

Dependent Variable: perceived impact of participation in the ARena project on decision-making on antibiotic prescribing;
