# Supplementary material for: Contextual factors influencing physicians’ perception of antibiotic prescribing in primary care in Germany — a prospective observational study
Source: BMC Health Serv Res. 2022 Mar 12;22:331. doi: 10.1186/s12913-022-07701-3 (PMC8917632; doi:10.1186/s12913-022-07701-3)
Supplement: Supplementary file 7 — Additional file 7. Results multilevel analysis. [file 12913_2022_7701_MOESM7_ESM.pdf]

Additional file 7

Results multilevel analysis

						Wald-CI 95%	
**Variables**	**B**	**SE**	**Wald**	**df**	**P value**	**lower**	**upper**
Constant	-3.425	0.671	26.051	1	0.000	-4.741	-2.11
Work experience	0.050	0.021	5.493	1	0.019	0.008	0.091
PCN environment	0.449	0.252	3.166	1	0.075	-0.046	0.944
Structural conditions	0.143	0.297	0.233	1	0.629	-0.438	0.725
Environment of existing processes	0.144	0.248	0.335	1	0.563	-0.343	0.631
External defined general conditions	0.201	0.299	0.450	1	0.502	-0.385	0.787

Dependent Variable: perceived impact of participation in the ARena project on decision-making on antibiotic prescribing
